# Loss of Farnesoid X Receptor Signaling Promotes Choroidal Neovascularization in Mice

**DOI:** 10.64898/2026.10.21.760483

**Published:** 2026-10-23

**Authors:** Chanel Harris, Mohammed A. Nisar, Ankit Seth, Allston Oxenrider, Briah Bailey, Gayatri Seth, Pamela M. Martin, Ravirajsinh N. Jadeja, Menaka C. Thounaojam

## Abstract

Choroidal neovascularization (CNV) is a defining feature of neovascular age-related macular degeneration (nAMD). Although anti-vascular endothelial growth factor (VEGF) therapies suppress neovascular activity, incomplete responses, recurrent leakage, and subretinal fibrosis remain important clinical challenges. The farnesoid X receptor (FXR) is a bile acid–activated nuclear receptor with anti-inflammatory, anti-fibrotic, and anti-angiogenic functions in other tissues, but its role in CNV is unknown. Analysis of publicly available human transcriptomic data (GSE135092) showed FXR expression in the retinal pigment epithelium (RPE)/choroid and significant downregulation in AMD-affected macular RPE/choroid. To define the role of FXR in CNV, laser photocoagulation was performed in wild-type (WT) and FXR-deficient (FXR ^−/−^ ) mice, with disease progression assessed by in vivo imaging, histology, *ex vivo* choroidal sprouting, and endothelial cell assays. FXR expression in the RPE/choroid was reduced 14 days after laser injury. Compared with WT mice, FXR ^−/−^ mice developed larger CNV lesions, more extensive hyperfluorescence on fluorescein angiography, and increased lesion area during disease progression. FXR deficiency also impaired resolution of fibrotic remodeling. In WT mice, α-SMA peaked at day 14 and declined by day 21, whereas collagen I remained low throughout; in FXR ^−/−^ mice, both collagen I and α-SMA were significantly elevated relative to WT and remained significantly higher than WT at day 21. Consistent with these findings, FXR- deficient choroidal explants exhibited increased vascular sprouting, and FXR ^−/−^ endothelial cells formed more extensive capillary-like networks. Together, these findings identify FXR as an endogenous regulator of pathological angiogenesis, vascular leakage, mesenchymal activation, and fibrotic remodeling during CNV and provide a rationale for investigating FXR-directed strategies for nAMD.

**Impact Statement:**

Neovascular age-related macular degeneration remains limited by recurrent vascular leakage and subretinal fibrosis despite effective VEGF suppression. This study identifies the bile acid-activated nuclear receptor FXR as a previously unrecognized regulator of the angiogenic and fibrotic responses underlying choroidal neovascularization. Integrating human AMD transcriptomic data with genetic, in vivo, ex vivo, and endothelial-cell approaches, we show that FXR expression is reduced in diseased RPE/choroid and that FXR deficiency enhances neovascular growth, vascular leakage, endothelial angiogenic activity, and persistent myofibroblast activation. These findings connect bile acid receptor signaling with RPE/choroidal vascular homeostasis and provide a biological foundation for evaluating FXR-directed approaches as complementary strategies for neovascular AMD.

## Full Text Availability

The license terms selected by the author(s) for this preprint version do not permit archiving in PMC. The full text is available from the preprint server.

